# Multilevel approach to plant–nanomaterial relationships: from cells to living ecosystems

**DOI:** 10.1093/jxb/erad107

**Published:** 2023-03-22

**Authors:** Halley Caixeta Oliveira, Amedea Barozzi Seabra, Selahattin Kondak, Oluwatosin Peace Adedokun, Zsuzsanna Kolbert

**Affiliations:** Department of Animal and Plant Biology, State University of Londrina (UEL), Londrina, PR, 86057-970, Brazil; Center for Natural and Human Sciences, Federal University of ABC (UFABC), Santo André, SP 09210-580, Brazil; Department of Plant Biology, University of Szeged, Szeged, 6726, Hungary; Doctoral School of Biology, Faculty of Science and Informatics, University of Szeged, Szeged, 6726, Hungary; Department of Plant Biology, University of Szeged, Szeged, 6726, Hungary; Department of Plant Biology, University of Szeged, Szeged, 6726, Hungary; University of Illinois, USA

**Keywords:** Mycorrhiza, nanomaterials, plant cell, plant growth-promoting rhizobacteria, pollinators

## Abstract

Due to their unique properties, nanomaterials behave peculiarly in biosystems. Regarding plants, the interactions of nanomaterials can be interpreted on a spatial scale: from local interactions in cells to systemic effects on whole plants and on ecosystems. Interpreted on a time scale, the effects of nanomaterials on plants may be immediate or subsequent. At the cellular level, the composition and structure of the cell wall and membranes are modified by nanomaterials, promoting internalization. The effects of nanomaterials on germination and seedling physiology and on the primary and secondary metabolism in the shoot are realized at organ and organism levels. Nanomaterials interact with the beneficial ecological partners of plants. The effects of nanomaterials on plant growth-promoting rhizobacteria and legume–rhizobia symbiosis can be stimulating or inhibitory, depending on the concentration and type of nanomaterial. Nanomaterials exert a negative effect on arbuscular mycorrhiza, and vice versa. Pollinators are exposed to nanomaterials, which may affect plant reproduction. The substances released by the roots influence the availability of nanomaterials in the rhizosphere, and components of plant cells trigger internalization, translocation, and transformation of nanomaterials. Understanding of the multilevel and bidirectional relationship between plants and nanomaterials is of great relevance.

## Teaser

The plant–nanomaterial relationship is bidirectional and can be interpreted on spatial and time scales. Beyond the direct effects on plants, nanomaterials affect beneficial ecological plant partners (e.g. rhizobacteria and pollinators).

## Introduction

Nanotechnology is a rapidly developing science, in engineering and technology, which has resulted in remarkable technical progress in recent years in sectors such as, *inter alia*, the cosmetic industry, food industry, and agriculture. This is due to the fact that nanotechnology produces nano-sized material with many beneficial properties (Joudeh and Linke, 2022). Nanomaterials (NMs) are characterized by surface area, surface charge, rate of agglomeration, particle morphology, and surface coating (Turan *et al.*, 2019). Even though they do not have a unified definition, nanoparticles (NPs) or NMs can be considered as particles with a diameter between 1 nm and 100 nm (Buzea *et al.*, 2007). Their ultra-small size lends them specific physicochemical properties that differ from those of bulk forms. NMs possess a large specific surface area, which provides them with high reactivity and physicochemical dynamicity (Mauter *et al.*, 2018). Further features of NMs include high surface energy and quantum confinement, which determine their behaviour and fate in different environments (Ma *et al.*, 2010). Thus, some authors consider that their unique properties compared with bulk materials are more relevant for the definition of NMs than applying strict size limits (Lee *et al.*, 2015).

NMs can originate from natural processes, such as activities of microbes, chemical precipitation, incomplete combustion, or geological effects, or can result from anthropogenic activities and intentional (e.g. nanopesticides and nanofertilizers) or unintentional release (garbage disposal, wear and corrosion, etc.) into the environment (Szőllősi *et al.*, 2020). NMs can be manufactured by chemical, physical, and biological methods. Biogenic approaches (green synthesis) using microbial (mainly bacterial and fungi) or plant material (e.g. algae, leaf extract, or flowers) have emerged recently as novel and eco-friendly forms of NM production (Ying *et al.*, 2022). The annual transition of synthetic NMs into the environment is considerably lower than that of natural NMs and is expected to release ~10.3 Mt year^–1^ into the atmosphere (Shukla and Iravani, 2017). Thus, sessile plants and their interacting partners (e.g. microbes or pollinators) come into contact with natural or engineered NMs in their environment.

Nowadays, there are hundreds of types of NMs, which, based on their composition, can be roughly categorized into two main groups: organic and inorganic NMs (Szőllősi *et al.*, 2020). Among the organic NMs, single- and multiwalled carbon nanotubes (CNTs) are frequently examined in relation to plants. CNTs have unique physical properties, such as thermal and electrical conductivity, elasticity, and resilience, and have been studied in plant science as growth promoters, carriers of genetic material, and potential agents with cellular and genetic toxicity (Velikova *et al.*, 2021). Furthermore, the effects of inorganic metal [mainly silver (Ag) and gold (Au) and metal oxide NPs [mainly zinc oxide (ZnO), titanium dioxide (TiO_2_), copper oxide (CuO), cerium(IV) oxide (CeO_2_), iron(II,III) oxide (Fe_2_O_4_), and iron(III) oxide (Fe_3_O_4_)] have been actively studied in diverse plant species. In general, beneficial, neutral, or detrimental effects of NMs have been documented in plants depending on the NM type, particle size, concentration, application method, treatment duration, target plant species, plant age, and experimental condition (Kah *et al.*, 2018; Kolbert *et al.*, 2022). Similar to this, NMs exert limited, negative, or even positive effects on soil microbial communities, functioning, and microbe–plant interactions (Tian *et al.*, 2019).

The goal of this review is to discuss the multilevel interaction of different NMs with plants, from the immediate, cellular NM effects through the effects on the whole plants, and to the later effects affecting living ecosystems. Furthermore, plants affect the characteristics and availability of NMs, creating a bidirectional interaction between plants and NMs. Additionally, we aim to draw attention to the fact that the effects of MNs in the environment go beyond the plants and extend also to the plants’ beneficial relationships with their living environment.

## Nanomaterials affect plants at the cellular and subcellular level

### Nanomaterials modify the structure of the plant cell wall, supporting internalization

At the cellular level, the first component that NMs have contact with is the cell wall. Plant cells are surrounded by a fibrillary macromolecule complex that dynamically adapts to changes in the environment. The cell walls can be characterized by their pore sizes due to the spaces between the fibrillary components within the matrix. According to the literature, the average pore size of the plant cell wall is 2–20 nm (Kurczyńska *et al.*, 2021), meaning that the cell wall performs a size exclusion task (size exclusion limit of most cell walls <10 nm). However, experimental data suggest that certain NMs are able to increase the cell wall pore size or trigger the formation of new pores by altering the chemical composition and thus penetrating into the apoplast.

Different NMs have been shown to increase or decrease the amount of most components in the wall (mainly pectin, hemicellulose, lignin, suberin, and callose; recently reviewed by Kurczyńska *et al.*, 2021; Wu and Li, 2022). NMs alter the amount of cell wall components by modulating the activity or expression of cell wall metabolic enzymes (e.g. xyloglucan endotransglycosylase/hydrolase, pectin methylesterase, phenylalanine ammonia lyase, cinnamyl alcohol dehydrogenase, 4-coumarate:coenzyme A ligase, and cell wall peroxidase) (Cui *et al.*, 2020; Madany *et al.*, 2020; de Almeida *et al.*, 2021; Guo *et al.*, 2022). Moreover, the NM-induced synthesis of reactive oxygen species (ROS) (mainly hydroxyl radicals) has been found to trigger cell wall loosening, allowing NMs to enter the cell wall (Kim et al., 2014, 2019; de Paiva Pinheiro *et al.*, 2021).

Intercellular movement of NMs in plants through apoplastic and symplastic routes seems to involve plasmodesmata (PDs). PDs have diameters of ~50 nm (Bell and Oparka, 2011), allowing the transport of NMs of this size range. The transport of various types of NMs (nAg, nPbS, SPION, nCeO_2_, nZnO, quantum dots, etc.) of different sizes (from a few nanometres to several tens of nanometres) through PDs has been suggested by several studies (reviewed by Molnár *et al.*, 2020; Kurczyńska *et al.*, 2021; He *et al.*, 2022; Labeeb *et al.*, 2022; Le *et al.*, 2022). What is more, the presence of 200–300 nm NPs has been observed in the apoplast and symplast of mustard plants (Preisler *et al.*, 2022a). It is hypothesized that the size exclusion limit of PDs may be altered by the presence of NMs due to mitochondria-associated oxidative stress; however, this possibility should be supported by further research (Kurczyńska *et al.*, 2021).

### NMs influence the composition of plant cell membranes

NMs that have passed through the cell wall interact with the plasmalemma as well as with intracellular membranes. The surface energy of NMs makes it difficult to pass through the cell membrane (Zhu *et al.*, 2012). There are several possibilities for NMs to pass through the membrane, including endocytosis, travelling via PDs, or causing physical disruption of the membrane. Clathrin-mediated endocytosis has an essential role for, *inter alia*, basic cellular functions, growth and development, and hormonal signalling (Chen *et al.*, 2011), and has been identified as a possible process of NM internalization into plant cells (Onelli *et al.*, 2008). Ultra-small NMs (<3 nm) may use membrane transporters or channels as entry points into the cell (Wu and Li, 2022). Moreover, in the case of high aspect ratio NMs, the mechanism of penetration through the membranes may be lipid exchange envelope penetration (Wong *et al.*, 2016). Quantum dots have been shown to form nanopores in lipid bilayers (Klein *et al.*, 2008) and are taken up by plant cells via fluid-phase endocytosis (Le *et al.*, 2022). These indicate that the pathway of interaction with plant membranes is determined by the type and the size of the NM. Additionally, the fact is not negligible that the membrane potential in plant cell membranes (–120 mV) is high enough to support the transport of NMs (Wu and Li, 2022).

For technical reasons, experiments on NM uptake have been performed mainly on protoplasts; thus, it is not known whether the above mechanisms also work in the case of plant cells in the tissue bundle. Recent results indicate that fullerene NMs increase or decrease the fluidity and permeability of the root cell membrane depending on the exposure time (He *et al.*, 2021). It is also evidenced that polyacrylamide-coated nanoceria decreases the activity and expression of lipoxygenase, thus protecting membranes against oxidative damage caused by salt stress (Li *et al.*, 2022). This suggests that NMs may influence the composition of plant cell membranes by altering fatty acid saturation and membrane-associated enzyme function.

Previous research has highlighted that NMs can be internalized into vacuoles, chloroplasts, and mitochondria (Milewska-Hendel *et al.*, 2019), raising the possibility that NMs also interact with organelle membranes at the subcellular level. Not much experimental evidence is available on NM transport into organelles but, due to the special composition and structure of organellar membranes, the mechanisms could be different depending on the organelle. It is considered possible that similarly to plasmalemma, the electrochemical gradient across the organelle membrane could be a driving force triggering NM transport (Wu and Li, 2022). NMs that enter the cell through the plasmalemma exert negative effects on organelles such as mitochondria, peroxisomes, vacuoles, and chloroplasts. NM-induced subcellular changes (e.g. swollen chloroplast structure, fewer thylakoids, abnormal size of plastoglobules and starch granules, destructive changes in peroxisomes, swollen mitochondrial cristae, and condensed nucleus) have been described (recently reviewed by Rajput *et al.*, 2019). Furthermore, the intracellular presence of NM can also affect the copy number of chloroplast and mitochondrial DNA (Pagano *et al.*, 2022), as well as the number and growth rate of the microtubule component of the cytoskeleton (Angelini *et al.*, 2022)

## NMs affect plants at the organ and organism levels

Following the immediate impacts of NMs at the cellular level, subsequent effects occur at the organ level. These effects on seeds, roots, and leaves affect the organism as a whole, with impacts on overall plant performance, as discussed in the subsections below.

### NMs influence seed germination and seedling growth

Seed germination and seedling establishment are critical stages of the life cycle of gymnosperms and angiosperms. Germinating seeds and seedlings are particularly sensitive to stresses, so that environmental and biotic factors may dramatically affect plant ontogenesis and stress responses, with consequences on plant survival, growth, and yield (Finch-Savage and Bassel, 2016; Pereira *et al.*, 2021; Kandhol *et al.*, 2022; Smolikova and Medvedev, 2022). Thus, these early stages of plant development are particularly prone to being affected by the interaction with NMs (Szőllősi *et al.*, 2020; H.M. Ahmad *et al.*, 2022).

The effects induced by NMs can be either negative (e.g. inhibition of germination, formation of abnormal seedlings, or phytotoxicity in seedlings) or positive (e.g. improvement in seed metabolism, increase in seedling vigour, or induction of stress resistance), depending on the concentration of the applied NM (Verma *et al.*, 2018; Khan *et al.*, 2019; Szőllősi *et al.*, 2020; A. Ahmad *et al.*, 2022), indicating that the effects of NMs on plant performance follow the concept of hormesis (Agathokleous *et al.*, 2019). Hormesis is characterized by a biphasic dose response, where stimulation occurs at low doses and inhibition occurs at high doses (Calabrese, 2014). The hormetic effect of NMs is also influenced by the characteristics of the NM and of the plant species (Khan *et al.*, 2019; Szőllősi *et al.*, 2020; A. Ahmad *et al.*, 2022). The composition of the NM, together with its charge, size, lipophilicity, and other features, have been shown to influence its interaction with seeds and seedlings (Nakasato *et al.*, 2017; Torrent *et al.*, 2020; Khalaki *et al.*, 2021; Kacsó *et al.*, 2022; Preisler *et al.*, 2022b). The rate of the hormetic response has also been shown to depend on whether the NM contains essential or non-essential nutrients. At the same time, element release from the NPs does not seem to be responsible for the beneficial effect of mild NP concentrations (Agathokleous *et al.*, 2019). Regarding the plant, traits related to seed size and anatomy, root architecture, and metabolism can influence the interaction with NMs and hence their actions (Rico *et al.*, 2011; Jain *et al.*, 2017; Verma *et al.*, 2018; Khalaki *et al.*, 2021). Environmental conditions, such as temperature, pH, light intensity, and exposure medium, may further alter the effects of NMs on plants (Verma *et al.*, 2018).

NMs tend to adhere to the seed tegument to a greater extent than the respective bulk material, which reinforces the relevance of the nano size scale for interactions with the seed (Li *et al.*, 2019). Depending on their characteristics, NMs can be internalized into the seed and transported by mass flow, mainly along the apoplast, in addition to being absorbed by the emerging radicle (Schwab *et al.*, 2016; Rico *et al.*, 2017; Savassa *et al.*, 2018; Preisler *et al.*, 2022b). When comparing NMs of similar types, smaller and negatively charged NMs usually penetrate more easily into the seeds (Cai *et al.*, 2017; Spielman-Sun *et al.*, 2019).

Water uptake and ROS homeostasis are crucial processes during seed germination and seedling growth that can be influenced by NMs (Shelar *et al.*, 2021; Nile *et al.*, 2022). Some types of NMs, such as CNTs, are able to promote the formation of pores in the seed coat and cell wall which, together with the induction of aquaporin expression, favour water and nutrient uptake by the seed and thus the germination process (Lahiani *et al.*, 2013; Tiwari *et al.*, 2013; Dasgupta-Schubert *et al.*, 2017). NMs may also induce aerobic respiration and generation of ROS, mainly hydrogen peroxide, which have important signalling functions for many processes crucial for triggering germination, including cell wall loosening, endosperm weakening, and cell elongation (Pereira *et al.*, 2021; Rai-Kalal *et al.*, 2021; Kandhol *et al.*, 2022). By favouring the interplay between hydrogen peroxide and gibberellins, the production of α-amylase is enhanced, which improves starch mobilization and soluble sugar release to support embryo and then seedling growth (Kandhol *et al.*, 2022). Not only is the seed primary metabolism modulated by NMs, but the production of secondary metabolites with signalling and/or antioxidant functions, such as flavonoids and oxylipins, is also affected (Kasote *et al.*, 2019; Samadi *et al.*, 2020).

In addition to the effects induced by NMs *per se*, some types of NMs (such as polymeric NPs) can be used as efficient carrier systems for bioactive molecules (e.g. plant growth regulators and metallic ions), which further regulate specific processes in germination (Pereira *et al.*, 2017; Gomes *et al.*, 2021). The beneficial effects triggered by NMs or molecules released by them are not restricted to germination, as improved seedling growth and stress resistance are observed, which can result in higher productivity (Pereira et al., 2019, 2021; Shelar *et al.*, 2021; Nile *et al.*, 2022).

**Fig. 1.**
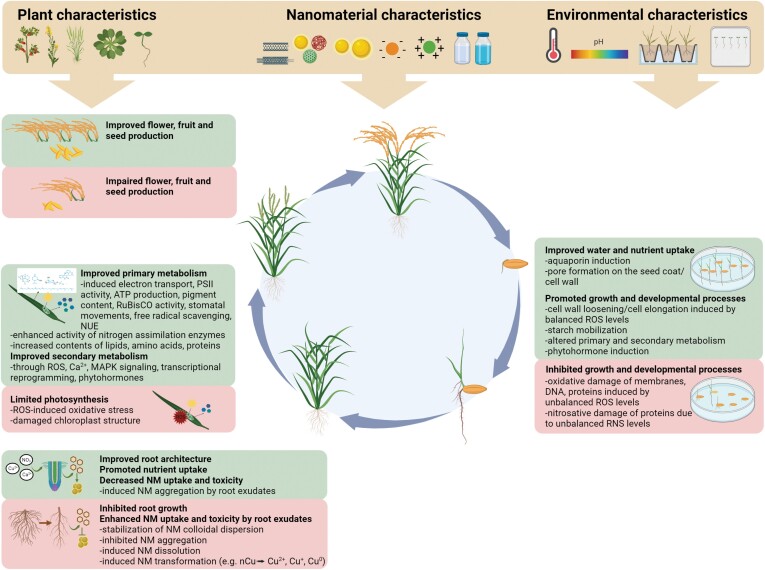
Nanomaterials (NMs) positively or negatively affect physiological processes during the whole life cycle of plants. The quality of the effects of NMs on plants depends on the characteristics of the plant (e.g. species, developmental stage), the NM (e.g. chemical nature, particle size, surface charge, concentration), and the environmental conditions (e.g. temperature, pH, growth medium). MAPK, mitogen-activated protein kinase; nCu, copper nanoparticles; NUE, nutrient use efficiency; RNS, reactive nitrogen species; ROS, reactive oxygen species.

These effects of NMs are usually accompanied by the induction of antioxidant enzymes, which keep ROS levels in the range of the ‘oxidative window’ for signalling functions (Kumar *et al.*, 2020; Chen *et al.*, 2021; Pereira *et al.*, 2021; Rai-Kalal *et al.*, 2021; Kandhol *et al.*, 2022). However, depending on the concentration and other characteristics of the NMs, oxidative damage resulting from high ROS levels might occur, yielding detrimental effects (e.g. loss of membrane stability and DNA damage), with reduced germination and seedling vigour (Szőllősi *et al.*, 2020; Pereira *et al.*, 2021; Nile *et al.*, 2022; Fig. 1). The phytotoxicity exerted by NMs can also be related to the disturbance of reactive nitrogen species homeostasis and the consequent induction of nitrosative stress in seedlings (Molnár *et al.*, 2020).

### NMs affect the root system

One of the major entry routes of NMs in plants is through the roots. For instance, it has been reported that ZnO NP uptake follows the order: root >leaf >stem in soybean (*Glycine max* L.; Yusefi-Tanha *et al.*, 2022), since the root is in direct contact with the soil (for terrestrial plants) or with water (for aquatic/floating plants). Moreover, ZnO NPs change the root architecture (root length, area, biomass, density, and volume) (Yusefi-Tanha et al., 2020, 2022). In fact, administration of ZnO NPs (up to 200 mg kg^–1^) to soybean roots had beneficial effects on root development and growth, whereas toxic effects were observed for higher concentrations (Yusefi-Tanha *et al.*, 2022). This indicates that similar to seedling growth, the root system-modulating effect of NMs also follows the concept of hormesis. Similar results were reported for CuO NPs, with different sizes (25–250 nm) and concentrations in soybean roots. CuO NPs altered root architecture depending on the NP size and concentration (Yusefi-Tanha *et al.*, 2020). Changes in root morphology can be assigned to a localized release of Cu^2+^ from NPs, upon NM contact with root surface cells, since Cu^2+^ can affect the epidermal cell differentiation and meristematic activity in roots (Yusefi-Tanha *et al.*, 2020). Thus, understanding the NM interactions with roots is of fundamental importance. Several parameters control the effects of NMs on roots, such as the chemical nature of the NM, surface, surface charge, size, morphology, agglomeration and aggregation state, plant species, etc.

A stable colloidal suspension of the NM tends to better interact with roots and be internalized. In contrast, large NM agglomerates tend to precipitate, decreasing the degree of positive interactions of the NM with the root tissue. Moreover, large NM aggregates make the passive transport of the material through cell wall pores in the plant roots difficult, since the average diameter of root cell wall pores is reported to be between 5 nm and 20 nm (Fleischer *et al.*,1999; Miralles *et al.*, 2012; Ye *et al.*, 2018). Similarly, NM surface charge influences the particle uptake by roots. Usually, due to the root epidermis cells, which have negatively charged polysaccharides, positively charged NPs are often more easily absorbed by roots, compared with negatively charged NPs.

Recently, single-layer graphene and few-layer graphene at a concentration of 50 g kg^−1^ increased maize growth by boosted nutrient absorption by maize (Wang *et al.*, 2023). Moreover, both tested graphene (single- and few-layer graphene) in the soil stabilized soil large agglomerates, while enhancing their content in the soil and inhibiting agglomerate disruption. Adding different layers of graphene into the soil dispersed soil particles to clump, forming water-stable agglomerate particles, which enhance crop growth and improved soil structure. Thus, NM features might impact soil constituents and properties.

The surface of NMs can be functionalized with active compounds to improve the positive interactions of NMs with plant tissue, including the root system, enhancing the ability of NMs to act as effective drug delivery systems of agrochemicals for practical applications. Moreover, chemical functionalization of NMs might improve the physicochemical properties of the NM, avoiding aggregation, and enhancing uptake and translocation (Kumar *et al.*, 2013). For instance, polycaprolactone and amine-functionalized iron oxide (Fe_3_O_4_) NPs altered NP uptake and translocation in tomato (*Solanum lycopersicum*), compared with non-functionalized NPs (Lau *et al.*, 2020). In a similar strategy, citric acid surface-modified hydroxyapatite NPs were used to increase phosphorus (P) uptake by *Zea mays* (as model crop) to overcome low P uptake by plants with current commercial P fertilizers (Samavini *et al.*, 2018).

Most plants absorb NMs by their roots, followed by translocation of the NMs to other parts of the plant, such as leaves and stems, and this process is driven not only by NM features (physicochemical and morphological parameters) but also by the plant species and the environment. Epidermal cells extend out in root hairs, enhancing permeation of the roots. NM absorption by roots is the first phase of NM accumulation and, once absorbed by roots, NMs can be translocated to other parts of the plants by apoplastic or symplastic routes. The first route involves the movement of dissolved minerals and water through cell walls, while the latter involves the solutes and water transport though the cytosol of neighbouring cells (Murali *et al.*, 2022). Depending on their size and agglomeration state, NMs can block root pores, impairing the apoplastic flow of micronutrients and water (Ruotolo *et al.*, 2018). Further details regarding root physiology and NM permeation can be found in Chichiriccò and Poma (2015), Ruotolo *et al.* (2018), and Murali *et al.* (2022).

The nature of the plant species is another important factor in the interactions between the NM and root. For example, if the plant is terrestrial or aquatic and, in particular, a floating plant, the root interactions with the exposed NM might be significantly different. Most of the studies based on NM interactions with plant roots are based on terrestrial plants, and studies on aquatic plants are still limited. In this direction, the interactions of CuO NPs with a typical floating duckweed species (*Lemna minor* L.) were reported (Yue *et al.*, 2018). CuO NPs were taken up by root cells, which were further distributed to plant fronds, indicating that the major entry of the NP into the aquatic plant was through the root. The internalized CuO NPs caused toxicity by generation of ROS and release of Cu^2+^ (Yue *et al.*, 2018).

Toxicity of NMs can be observed due to particle internalization, accumulation, and generation of ROS, along with the release of metal ions, which can interact with biomolecules (Coman *et al.*, 2019) (Fig. 1). Interestingly, NMs can affect plants only by their direct contact with roots, without being absorbed and translocated. It has been reported that negatively charged Au NPs can impair the root development of barley (*Hordeum vulgare* L.), by their contact with the root, with no evidence of NP internalization (Milewska-Hendel *et al.*, 2022).

It can be difficult to predict NM uptake and translocation by roots, since several factors need to be considered, including the properties of the NMs, environmental conditions, and application methods. Indeed, it is a challenge to track NM interactions with roots since, once released into the environment, NMs undergo transformations, which can facilitate or not their accumulation in soil and uptake by roots (Rajput *et al.*, 2020; Murali *et al.*, 2022). Thus, NM interactions with roots can be variable with respect to several parameters, which might further impact the positive or negative effects of NM application in plants.

### NMs affect metabolic processes in the shoot

NMs may reach the shoot via xylem flow when applied to the root (Lin *et al.*, 2009; Ye *et al.*, 2018; Preisler *et al.*, 2022a). After foliar application, NMs can penetrate the leaf through the cuticle, stomata, hydathodes, trichomes, lenticels, and wounds (Avellan *et al.*, 2019; Bombo *et al.*, 2019; Khan *et al.*, 2019). Inside the leaf, NMs may access the mesophyll through apoplast or symplast pathways, eventually reaching the phloem for long-distance translocation (Avellan *et al.*, 2021).

Photosynthesis is one of the main processes of plant physiology and primary metabolism that can be affected by NMs in the leaves. Studies have demonstrated the potential of some carbon-based NMs (mainly CNTs and carbon dots) for enhancing photosynthesis (Swift *et al.*, 2018). It has been proposed that these NMs augment CO_2_ assimilation by different mechanisms, which include the increase in electron transport rate, PSII activity, ATP production, photosynthetic pigment levels, and Rubisco carboxylase activity, as well broadening of the light absorption range (Swift *et al.*, 2018; Ghorbanpour *et al.*, 2021; Li *et al.*, 2021; Ranjan *et al.*, 2022).

Carbon dots have been more commonly used to promote photosynthesis as they show lower toxicity and less complex synthesis than CNTs (Swift *et al.*, 2018). However, the induction of ROS production by carbon-based NMs may prevent the translation of enhanced photosynthesis into increased plant growth (Swift *et al.*, 2018; Milenković *et al.*, 2021). Thus, functionalized carbon dots have been developed as a strategy to augment photosynthesis without leading to oxidative stress, having positive impacts on crop yield (Swift *et al.*, 2021).

In addition to carbon-based NMs, other types of NMs have been shown to modulate photosynthesis. For example, fluorescent, amine-functionalized silicon quantum dots (2.4 nm) were used as artificial antennas to amplify the light harvesting ability and improve the photosynthesis of Italian lettuce (Li *et al.*, 2020). Additionally, chitosan NPs can augment photosynthesis though the modulation of stomatal aperture, chlorophyll content, antioxidant response, and nutrient use efficiency (Kumaraswamy *et al.*, 2018). In the case of metal/metal oxide NMs, the stimulatory effects of TiO_2_ NPs on photosynthesis must be highlighted, due to their capability to increase light absorption (particularly UV light) and protect chlorophyll molecules (Ghorbanpour *et al.*, 2021; Ranjan *et al.*, 2022). Other metal-based NMs (e.g. Au, manganese oxide, and Fe NPs) can promote photosynthesis through different mechanisms, such as the scavenging of free radicals, increase in chlorophyll content, and promotion of electron transfer (Wang *et al.*, 2020; Ghorbanpour *et al.*, 2021; A. Ahmad *et al.*, 2022). It is noteworthy that, in general, all types of NMs might negatively affect photosynthesis depending on the applied concentrations, due to the induction of oxidative stress and damage to chloroplast structures (Ghorbanpour *et al.*, 2021; A. Ahmad *et al.*, 2022; Fig. 1). This means that photosynthetic processes present a hormetic response to the presence of NMs.

NMs may also affect pathways of plant primary metabolism other than photosynthesis by modulating the biosynthesis and activity of key enzymes (Hatami *et al.*, 2016; Bouyahya *et al.*, 2022). For example, TiO_2_ NPs have been shown to increase the activity of nitrogen (N) assimilation enzymes (e.g. nitrate reductase and glutamine synthetase) of spinach plants (*Spinacia oleracea* L.), yielding higher levels of proteins and chlorophylls, as well as improved biomass accumulation (Yang *et al.*, 2006). Both up- and down-regulation of carbohydrates, lipids, amino acids, and proteins have been reported after the treatment of plants with NMs, depending on the plant species and NM characteristics/concentration (Hatami *et al.*, 2016; Bouyahya *et al.*, 2022).

In addition to primary metabolism, NMs induce the production of secondary metabolites of different classes (alkaloids, phenolics, and terpenoids), which are involved in the plant acclimation to adverse environmental conditions and in biotic interactions (Anjum *et al.*, 2019). Although not fully elucidated, the elicitation mechanisms seem to involve ROS production, Ca^2+^ fluxes, mitogen-activated protein kinase cascades, transcriptional reprogramming, and interaction with phytohormones (Marslin *et al.*, 2017; Anjum *et al.*, 2019; Khan *et al.*, 2021).

By modulating a wide array of signalling and metabolic processes in the shoot, NMs may have important effects on different processes of plant vegetative and reproductive development. Studies with various types of NMs (including carbon NMs, metallic/metal oxide NPs, and polymeric NPs) have reported positive effects of these nanostructures on the production of flowers, fruits, and seeds of many plant species, resulting in increased yield (Jaberzadeh *et al.*, 2013; Khodakovskaya *et al.*, 2013; Tarafdar *et al.*, 2014; Pereira *et al.*, 2019; Phong *et al.*, 2022). For example, Gupta *et al.* (2022) demonstrated that the foliar application of ZnO and F_3_O_4_ NPs to cucumber (*Cucumis sativus* L.) plants increased the seed yield and quality. In a study with litchi (*Litchi chinensis* Sonn.), TiO_2_ NP spraying improved the pollen germination rate and pollen tube length, as well as the fruit set rates (Y. Huang *et al.*, 2022). Moreover, the promotion of early flowering has been reported for chitosan NPs, ZnO NPs, and cadmium sulfide quantum dots (Prasad *et al.*, 2012; Marmiroli *et al.*, 2020; Shafiei-Masouleh, 2022), as well as the induction of shoot branching by CNTs (Tripathi *et al.*, 2011).

Another specific process of shoot development that can be affected by NMs is the senescence of fruits, flowers, and vegetative organs. In this case, studies have focused on the application of NMs which release bioactive compounds that have a well-described anti-senescent effect, such Ag-based NPs and polymeric NPs loaded with nitric oxide donors (Ma *et al.*, 2019; Naing and Kim, 2020).

As observed for other physiological processes, treatments with NMs may lead to undesirable, deleterious effects on shoot development, depending on characteristics related to the plant and the NM (Verma *et al.*, 2018; Fig. 1). A good example comes from the study by Salehi *et al.* (2021), who showed that the foliar application of CeO_2_ NPs at high concentrations induced deleterious effects on pollen grain and ovule formation in bean crops (*Phaseolus vulgaris* L.), in spite of the amelioration of plant productivity at optimal dosages. Since low NM doses stimulate, while high doses inhibit the vegetative and reproductive growth of the shoot, the plant response to NMs can be considered as hormetic. Another example of negative effects of NMs on plant reproduction is the reduction of pollen adhesion caused by the exposure of flowers of wind-pollinated plants to graphene-related NMs (Zanelli *et al.*, 2022). Moreover, Zhao *et al.* (2015) observed that ZnO NP exposure significantly decreased the number of fully developed cobs in corn plants, suggesting that nZnO inhibits flower fertilization or pollination.

## NMs affect beneficial interactions of plants with their living environment

Mainly through their root and shoot system, plants may establish beneficial relationships with the organisms in their environment, including microorganisms and more complex living beings (such as pollinating insects and mammals). These mutually positive relationships play important roles in plant ecophysiology. According to the research data accumulated to date, the presence of NMs primarily affects the interactions of plants with beneficial bacteria, fungi, and pollinating insects.

### The effects of NMs on the plant microbiome

As part of the living environment, the microbiome or microbiota is the diversity of microorganisms (viruses, bacteria, fungi, and oomycetes) with which plant organs are associated. Representatives of the microbiota can be endogenous or surface-living microorganisms, and their presence generally has a beneficial effect on plant growth and stress resistance (Chialva *et al.*, 2022). The most commonly studied microbiota components in terms of NM effects are the plant growth-promoting rhizobacteria (PGPR), including legume–rhizobia symbiosis and arbuscular mycorrhiza.

#### The effect of NMs on PGPR–plant interactions

PGPR are free-living bacteria that can colonize in the rhizosphere, rhizoplan, or root tissues, thus establishing an association with plant roots. These bacteria induce plant growth via different mechanisms, such as the production of phytohormones, siderophores, antioxidants, exopolysaccharides, osmoprotectants, and enzymes, enhancing nutrient uptake and intensifying plant stress resistance against abiotic and biotic factors (Abdelaal *et al.*, 2021; Mishra *et al.*, 2021).

Among NMs, most of the data relate to the effect of different metal oxide NPs, as recently reviewed by de Moraes *et al.* (2021). Cu, Zn, silicon (Si), Ti, and Au NPs have been shown to increase the number and viability of bacterial cells and improve the beneficial effects of PGPR, such as plant growth induction, hormone production, and siderophore release (de Moraes *et al.*, 2021, and references therein). The majority of early studies (Dimkpa *et al.*, 2012; Karunakaran *et al.*, 2013; Rangaraj *et al.*, 2014; Shukla *et al.*, 2015) did not investigate the consequences of NM exposure on the plant growth-inducing effect of PGPR. However, these studies observed that the positive or negative nature of the effect on PGPR depends on the NM characteristics and concentration, the bacterial strain, and the duration of the treatment. Due to the concentration-dependent effect of PGPR on bacteria, the response to NMs appears to be hormetic.

An interesting study demonstrated the direct effect of TiO_2_ NPs on the association of oilseed rape (*Brassica napus* L.) roots and PGPR; it was detected that TiO_2_ NPs increase the ­adhesion of beneficial bacteria to the roots and protect the plants against fungal infection (Palmqvist *et al.*, 2015). More recent research examined the combined effect of NM and PGPR treatment on plant physiology, providing insight into the influence of NMs on the beneficial effects of PGPR. For instance, in cucumber, Ag NPs inhibit the effect of PGPR on root and shoot length, but intensify the protein and phenolic contents of leaves (Nawaz and Bano, 2020). In their study, Merinero *et al.* (2022) aimed to improve the Fe biofortification potential of Fe NPs in wheat (*Triticum aestivum* L.) by applying PGPR (*Bacillus aryabhattai* RSO25), but the combined treatment with PGPR and Fe NPs caused intermediate Fe accumulation in spikes compared with the single treatments, indicating antagonism between polymeric Fe NPs and PGPR (Merinero *et al.*, 2022). In wheat exposed to arsenic stress, green-synthetized molybdenum (Mo) NPs stabilized *Bacillus* sp. ZH16 and improved phytobeneficial traits in the bacterium (H.M. Ahmad *et al.*, 2022). Moreover, combined treatment with the biogenic Mo NPs and the ZH16 strain remodelled ionic/nutrient profiles and mitigated arsenic toxicity in wheat (H.M. Ahmad *et al.*, 2022).

**Fig. 2.**
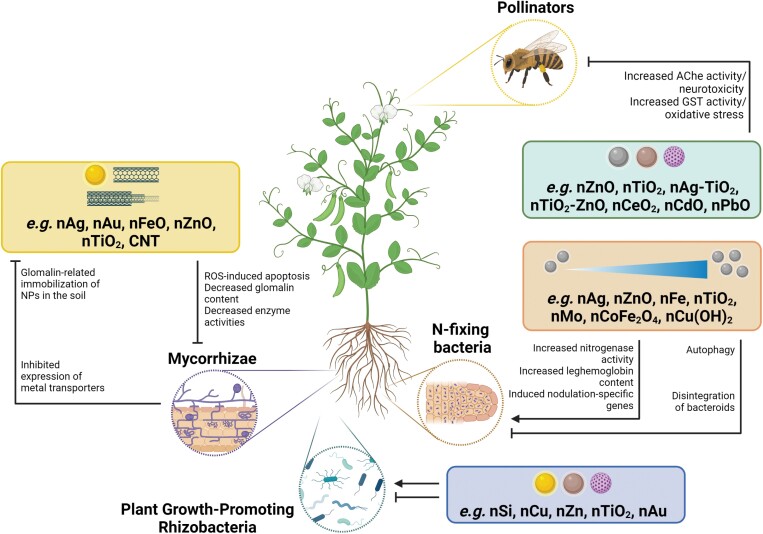
The interaction between nanomaterials (NMs) and beneficial ecological partners of plants. NMs can positively or negatively affect plant growth-promoting rhizobacteria. Depending on their concentration, NMs promote or limit N-fixing symbiosis. Mycorrhization may alleviate NM-induced stress in plants but, conversely, the presence of NMs may negatively influence the physiology of mycorrhizal fungi. Diverse types of NMs cause neurotoxicity and oxidative stress in honey bees as representative plant pollinators. NPs, nanoparticles; ROS, reactive oxygen species; AChe, acetylcholinesterase; GST, glutathione *S*-transferase.,

All of the above indicate that the presence of NMs can influence the PGPR–plant relationship positively or negatively, meaning that there can be synergistic or antagonistic relationships between NMs and PGPR (Fig. 2).

A special case of the PGPR–plant relationship is the symbiosis of many legume species with rhizobia. Biological fixation of atmospheric nitrogen (N_2_) is essential for the maintenance of life on earth (Stevenson, 1982). Only a few genera of prokaryotes contain the genetic information for synthesizing nitrogenase enzyme, that catalyses the conversion of gaseous N_2_ into ammonia, which can then be utilized for the synthesis of organic N compounds (Pate, 1980; Giller, 2001; Ladha *et al.*, 2022).

Most of the data available on the effect of NMs on symbiosis are related to metal-based NPs (e.g. Ag, ZnO, Fe, TiO_2_, and Mo NPs), which have recently been reviewed by Ameen *et al.* (2021). In most cases, the presence of NM negatively affects nodulation, through processes such as morphological alterations in rhizobial cells and a damaged bacterial surface. Furthermore, NMs may cause early nodule senescence and delayed N fixation. The presence of NMs may also result in the intracellular deterioration of cytoplasmic components due to autophagy and disintegration of bacteroids. In some cases, the presence of NMs is neutral to the symbiosis, and in some cases it stimulates nodulation. The NM concentration determines the effect on nodulation, as demonstrated in the case of nanoCu(OH)_2_ and CeO_2_ NPs during *Phaseolus vulgaris*–*Rhizobium leguminosarum* and soybean–*Bradyrhizobium japonicum* symbioses, respectively (Baijukya and Semu, 1998; Priester *et al.*, 2012).

Recent results confirmed that the effect of NMs on legume–*Rhizobium* symbiosis can be highly diverse. Intensifying the positive effect of elevating concentrations was demonstrated in the case of Ag NPs in soybean, manifested through a significant increase in the number of root nodules, an increase in soybean yield, and enhancement in the enzymatic activity of peroxidases and polyphenol oxidases (Krutyakov *et al.*, 2022). A beneficial effect on nodulation was also observed in the case of Fe_3_O_4_ NPs in common bean, soybean, and alfalfa (*Medicago sativa* L.) due to increased nitrogenase activity and haemoglobin content (De Souza-Torres *et al.*, 2021; Groppa *et al.*, 2022). Cobalt ferrite (CoFe_2_O_4_) nanozyme with antioxidant properties effectively reduces the concentration of ROS in the nodule, creating an ideal environment for the proliferation of rhizobia and forming more effective nodules. Furthermore, the CoFe_2_O_4_ nanozyme proved to be a synergist of leghaemoglobin and increased its accumulation (Ma *et al.*, 2021). Transcriptome sequencing performed in soybean exposed to manganese ferrite NPs identified 31 differentially expressed genes associated with soybean nodulation that were up-regulated in the late inoculation stage, resulting in improved N fixation efficiency (Ma *et al.*, 2022). At the same time, a concentration-dependent effect on N-fixing symbiosis was detected in ZnO NP-treated alfalfa, where a lower ZnO NP dosage has a favourable effect, while a high concentration decreases the N-fixing area of root nodules, and the number of bacteroids and root nodules, which in turn adversely affects the N-fixing ability of alfalfa. A high concentration of ZnO NPs supresses the relative abundance and diversity of the soil microorganisms, drawing attention to the fact that in the short term, exposure to high-dose ZnO NPs damages the soil environment and the plant (Sun *et al.*, 2022). According to the latest results, CuO NPs induced the nitrogenase activity and the diazotrophic diversity in the soil, and they also caused a shift in the taxonomic and phylogenetic community composition in the rhizosphere of maize (Cao *et al.*, 2023). At the same time, the presence of CuO NPs in the rhizosphere destabilized the soil diazotrophic community and caused decreased plant biomass, indicating that the presence of CuO NPs in the maize rhizosphere is detrimental for both the soil biological functions and the plants (Cao *et al.*, 2023). Furthermore, the application of different varieties of soybean rhizosphere microorganisms (RMs) ameliorates the toxic effects of Mo NPs on plant growth and N fixation, suggesting that the administration of RMs can be a promising strategy to prevent the phytotoxic effects of NMs (Zhou *et al.*, 2023). Recently, molybdenum disulfide nanosheets have been shown to disturb nutrient uptake, growth, and physiology of maize seedlings as well as to induce changes in the composition of the soil bacteria community, favouring the enrichment of N-fixing bacteria (Chen *et al.*, 2023).

Based on the results so far, different types of NMs at low doses stimulate N-fixing symbiosis, thus increasing legume yield due to the activation of nitrogenase, the creation of an anaerobic microenvironment suitable for the enzyme, and the differential expression of nodulation-specific genes. The negative effect of higher NM dosages on symbiosis is the consequence of a damaged bacterial surface, intracellular deterioration of cytoplasmic components by autophagy, and decomposition of bacteroids (Fig. 2). These indicate that not only the direct effects of NMs on plant physiology, but also their indirect effects on the N-fixing symbiosis follow the hormesis concept.

#### The effect of NMs on mycorrhizal symbiosis

Arbuscular mycorrhiza (AM) is a symbiosis between plant roots and fungi of the phylum Glomeromycotina. This interaction is mutually positive since the extended hyphal network of the fungi absorbs nutrients (mostly phosphate, ammonium, nitrate, sulfate, and potassium) from the soil, and provides them to the plant, while the plant provides the fungi with carbohydrates and lipids (Berger and Gutjahr, 2021). Beyond nutrient supply, AM improves plant resistance against abiotic and biotic stressors, such as drought, salinity, or heavy metal exposure and pathogen attack.

It is evidenced that NMs accumulated in the soil affect the symbiosis between plants and AM fungi (AMF). In addition, there is a two-way interaction between AMF and NMs, since data support that AMF symbiosis improves the resistance of the plant partner in the case of NM-induced stress, and the presence of NMs in the rhizosphere affects the AMF plant adversely or beneficially by negative or positive feedback mechanisms (reviewed by Tian *et al.*, 2019; Ameen *et al.*, 2021) (Fig. 2).

The fungi affect the uptake, transport, and transformation of NMs in the soil–plant system due to the secretion of glomalin-related soil protein, which immobilizes metal NPs in the soil, thus reducing their bioavailability. However, this effect was not shown in all experimental systems, which suggests that the effect of AMF on the uptake of NPs depends on the plant species and metal type. The presence of AMF colonization inhibits the expression of membrane transport genes and, as a result, in the case of metal NPs, the absorption of metal ions into the plant is inhibited or reduced (Wang *et al.*, 2022, and references therein). According to Wang *et al.* (2022), AMF improves the resistance of the host plant against NP-induced stress by ameliorating oxidative stress, promoting soil enzyme activity, strengthening nutrient conversion by the rhizosphere, and extending the nutrient acquisitive range of plant roots.

Recently, the alleviating effect of AMF on Cu NP [Cu(OH)_2_ nanowires] toxicity was observed in spearmint (*Menta spicata* L.) (Apodaca *et al.*, 2022). Similar to this, CuO NP stress was ameliorated by AMF colonization in *Canna indica* L. plants due to the reduction in ROS production and the induction of the antioxidant defence at the gene level (Luo *et al.*, 2022). Furthermore, AMF inoculation decreased Cu levels in seedlings, possibly due to the increased expression of organic acid metabolism-associated genes and, consequently, intensified organic acid secretion (Luo *et al.*, 2022). In another system, the combined effect of AMF and NMs on wheat drought resistance was studied, revealing that Fe NPs combined with *Glomus intraradices* resulted in maximum wheat growth and yield as a consequence of intensified rhizosphere colonization, increased water use efficiency, and photosynthetic rate under drought stress. Furthermore, Fe NPs were shown to significantly enhance the growth-and drought resistance-inducing effects of *Glomus intraradices* (Naseer *et al.*, 2022). A synergistic effect has been demonstrated between MNs (nanoscale zerovalent iron, nZVI) and AMF in the case of drought-exposed maize, where next-generation sequencing showed that an appropriate amount of nZVI promotes the colonization and development of *Funneliformis mosseae* as the dominant species in the rhizosphere (Yang *et al.*, 2022). Moreover, AMF effectively immobilizes Fe NPs outside the roots, thus alleviating the adverse effects of excess metal (Yang *et al.*, 2022).

The NMs in the rhizosphere have been well documented to negatively affect the symbiosis between the AMF fungus and the plant root. Tian *et al.* (2019) and Ameen *et al.* (2021) recently reviewed the diverse negative effects of different NMs (e.g. nAg, nAu, nFeO, nZnO, nTiO_2_, and CNTs) on different AMF–plant systems. Physicochemical properties (e.g. type, speciation, size, and coating) are the main determinants in the impact of NMs on mycorrhiza colonization (Tian *et al.*, 2019; Wang *et al.*, 2022). Considering the size of the NM, it can influence the bioavailability and mobility of NMs in the soil and plant and consequently affect NM toxicity on AMF. Among all factors, the most influential is the concentration of NM in the growth medium. In several cases, low NM concentrations have no effect, while elevated doses have a negative impact on mycorrhizal symbiosis (Tian *et al.*, 2019). However, according to a recent study, low nZVI dosages promoted, while higher dosages inhibited AMF colonization as well as maize growth, suggesting that the impact of the NM on plant–mycorrhiza symbiosis is hormetic (Yang *et al.*, 2023). The results show that NMs change the morphology of the hyphae and induce apoptosis through ROS accumulation, loss of cytoplasm, cell wall damage, inhibition of enzyme activities (e.g. manganese peroxidase or laccase), and decreased glomalin production in the fungal partner (Wang *et al.*, 2022; Yang *et al.*, 2023). A further interesting study revealed that carbon NMs (reduced graphene oxide, multi-walled CNTs, and fullerene) alter the composition of fungal endophyta of rice, reduce the amount of the hormones indole-3-acetic-acid, zeatin riboside, and gibberellic acid, and inhibit plant growth. This research draws attention to how the fungus–plant symbiosis is disturbed as the result of CNT exposure and this symbiosis is sensitive even to low concentrations of CNTs (Hao *et al.*, 2020).

### The effects of NMs on plant pollinators

The plant–pollinator relationship is considered to be an important contributor to biodiversity on Earth. The overwhelming majority of flowering plants (~80%) are pollinated by animals (Ollerton *et al.*, 2011), and without pollinators more than half would suffer a notable reduction in seed yield (Rodger *et al.*, 2021). Insect pollinators (e.g. honey bees, ants, mosquitoes, and butterflies) contribute to increasing agricultural productivity by pollinating crops for seed and fruit set productivity (Kannan *et al.*, 2020). Pollinators such as honey bees have been adapted to collect pollen, but also unintentionally take other particles (e.g. NMs) to the hive, where these may accumulate (Hooven *et al.*, 2019). Therefore, studies have focused mainly on the effects of different NPs on honey bees.

In nature, NMs deposited from the atmosphere onto above-ground plant parts, such as leaves and flowers, may come into contact with pollinators, mainly due to surface exposure, inhalation, and foraging (Kos *et al.*, 2017). Furthermore, numerous studies have shown that NMs taken up through the root are transported in the vascular system to above-ground plant parts (e.g. López-Moreno *et al.*, 2010; D. Huang *et al.*, 2022). ZnO NPs or Zn ions in the form of ZnCl_2_ solution caused neurotoxicity in honey bees (indicated by a decrease in survival, and loss of brain weight and protein content) and a significant elevation in acetylcholinesterase (AChe) and glutathione *S*-transferase (GST) activities (Milivojević *et al.*, 2015). With increasing ZnO concentrations, the consumption rate declines, but honey bees prefer ZnO NM-containing food, even with the highest Zn doses, compared with the control diet. This indicates that honey bees might be able to perceive the presence of ZnO NMs in sucrose solution (Glavan *et al.*, 2017).

Increasing toxicity with elevating treatment concentrations and exposure duration was described for Ag–TiO_2_, ZnO–TiO_2_, and TiO_2_ NPs in honey bees (Özkan *et al.*, 2014). The time-dependent intensification of the toxic effect on honey bees was also confirmed in the case of boron NPs (Daǧlioǧlu *et al*., 2015). In another study, CeO_2_ NPs exposure increased AChe activity in the head and thorax of bees, which reflects a neuronal effect, as well as increasing GST activity, indicating intensified protection against oxidative stress. The majority of the observed effects were due to the CeO_2_ NPs, since the level of Ce ion species in the food was negligible (Kos *et al.*, 2017).

Furthermore, nano formulation of hexanal was tested at different concentrations on worker honey bee species (*Apis cerana indica*, *A. mellifera*, and *A. florea*) and was found to be ­harmless, with minimum mortality ranging from 4.05% to 7.65% (Mohan *et al.*, 2017). Recently, a decreased survival rate and feeding willingness were determined in bees fed with sugar syrup containing cadmuim oxide (CdO) NPs or CdO plus lead oxide (PbO) NPs. NP treatments promote the expression of antioxidant genes (e.g. GST, superoxide dismutase, and catalase), suggesting activated protection against oxidative stress, while enhanced AChe activity in the heads of honey bees reflects neurotoxic effects (Al Naggar *et al.*, 2020; Fig. 2). In an interesting recent study, sublethal doses of CdO NPs have been shown to disturb visual capabilities of the *Drosophila melanogaster* model which could also have long-term detrimental repercussions for, for example, pollinators due to the reduction of their efficacy in plant pollination, thus risking ecosystem functionality and food security (El Kholy and Al Naggar, 2023).

## Plants affect the characteristics and availability of NMs

The relationship between NMs and plants can be considered as bidirectional, since plants also affect NMs found in their environment. The NM-modifying effect of plants is due to root exudates and to substances in plant cells (e.g. thiols or nitrates).

### *Ex planta* transformation of NMs driven by root exudates

NMs can be modified by plants, mainly in the rhizosphere by root exudates, which can strongly interfere with NM uptake and translocation to other plant tissues. Positive or negative effects of NMs, including toxicity, can be attributed to their dissolution/aggregation in the environment (Shang *et al.*, 2019; Cervantes-Avilés *et al.*, 2021, Fig. 3). The NM transformation in agricultural environments is still not fully known. NMs can be affected in the rhizosphere by the soil and by the plant (Peng *et al.*, 2019; Shang *et al.*, 2019).

**Fig. 3.**
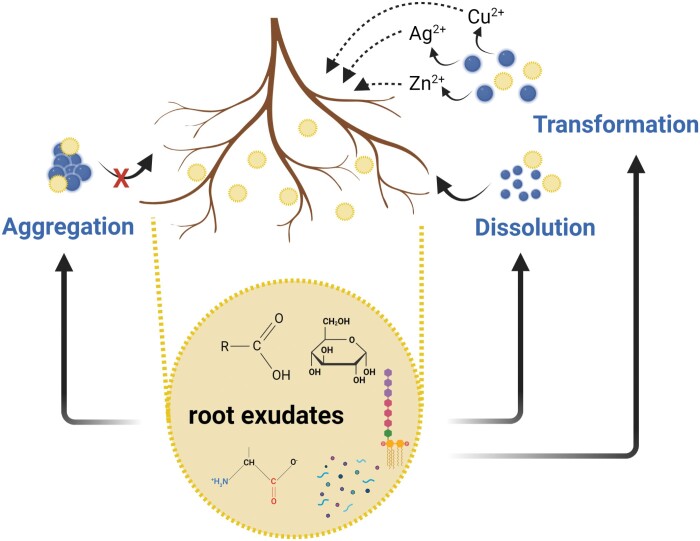
Root exudates induce aggregation, dissolution, and transformation of nanomaterials (NM)s in the rhizosphere. Aggregation inhibits, while dissolution favours, the plant uptake of NMs. Root exudate-induced transformation typically occurs in the case of metal-containing NMs which results in metal ion (e.g. Zn^2+^, Cu^2+^, and Ag^2+^) release. The ions are easily absorbed by plant roots.

Root exudates play a key role in the transformation of NMs. Root exudates can be very complex, since they can contain from low molecular weight substances (such as fatty acids, amino acids, organic acids, and monosaccharides) to high molecular weight substances (proteins and polysaccharides). Moreover, the external environment, such as nutrient deficiency or other abiotic stress conditions in the soil, can trigger the release of specific organic compounds from the roots (Dong *et al.*, 2004; Vranova *et al.*, 2013; Zhao *et al.*, 2016; Huang *et al.*, 2017; Peng *et al.*, 2019; Shang *et al.*, 2019). Indeed, the chemical nature of root exudates should be considered when addressing NP uptake and distribution by roots, since NMs interact positively or negatively with root exudates in the rhizosphere (Ye *et al.*, 2018). Root exudates can promote NM dissolution, transformation—which can positively or negative influence NM uptake—and accumulation (Huang *et al.*, 2017).

The bioavailability of NMs depends on the presence and nature of soil organic matter. In this sense, amino acids are major constituents of low molecular weight root exudates (Zhang *et al.*, 2015; Ye *et al.*, 2018), and NM stability in colloidal dispersion is affected by amino acids (Schwaminger *et al.*, 2015; Ye *et al.*, 2018). For instance, Ye *et al.* (2018) investigated the uptake of Au NPs coated with either positively or negatively charged molecules, in the presence or absence of the amino acids Lys or Asp by rice. These amino acids are the major constituents of rice root exudates. Interestingly, the presence of Lys or Asp interfered with the interactions (uptake and distribution) of Au NPs by rice roots, due to the electrical interaction between the coated NP and the amino acid. A positive interaction of the root tissue with Au NPs was observed with the same electrical charge of the Au NP surface and the amino acid, facilitating NP dispersion in the nutrient medium, and thus, increasing root uptake of Au NPs (Ye *et al.*, 2018).

It can be difficult to predict how NMs will interact with roots. For instance, in an interesting approach, low molecular weight organic acids were identified in rice root exudates, and the individual exudate was applied to CuO NPs. The authors demonstrated that either the nature or the concentration of low molecular weight organic acid exudate strongly interfere with CuO NPs aggregation, dissolution, and sedimentation (Peng *et al.*, 2019). Similarly, NP agglomeration was found when the charge of the NP coating was different from the charge of the amino acid, decreasing Au NP uptake by root tissue (Ye *et al.*, 2018). It should be noted that the initial surface charge of the NM is not the only important factor in the interaction and uptake of the NM by root, as the chemistry surface after NM interaction with environmental parameters, such as root exudate, plays a role in the uptake of the NM by roots.

### *In planta* biotransformation of NMs

After internalization, NMs undergo physical or chemical transformation, including aggregation, redox dissolution, sulfidation, chlorination, and complexation with organic matter, such as thiolates (D. Huang *et al.*, 2022). During aggregation, larger particles may be formed, while complexation results in the formation of a corona, consisting of mainly proteins or plant metabolites such as flavonoids and lipids. Bio-corona formation has been observed, *inter alia*, in the case of Ag NPs, CuO NPs, and TiO_2_ NPs in various plant species (Miclăuş *et al.*, 2016; Kurepa *et al.*, 2020; Borgatta *et al.*, 2021). Additionally, following internalization, NMs may transform into various species and, depending on the type of the NM, these can be phosphates, sulfates, thiols, nitrate, or acetate complexes, or ionic forms (Peng *et al.*, 2015). The aforementioned results mean that the presence of NM not only affects plant structure and function, but that the properties of NMs can also be affected within the plant cells/tissues.

## Application of theoretical knowledge about plant–NM interactions

The practical utilization of the accumulated theoretical knowledge regarding plant–NM interactions is a desirable goal. Newer nano-based procedures are being developed, for use in areas such as plant genetic transformation, sensor development, priming, nutrition, and phytostimulation, thus presenting huge potential to improve plant production for different goals (Aguirre-Becerra *et al.*, 2022; Bora *et al.*, 2022; Chakraborty *et al.*, 2023; Guleria *et al.*, 2023).

A promising field of NM application is in genetic transformation, because NPs can passively enter the cell wall and plasma membrane, have high affinity for binding DNA, and high transformation efficiency, without integrating the genome (Izuegbunam *et al.*, 2021). For the development of novel nano-biomimetic transformation systems, knowledge of the interactions of NMs with the cell wall and cell membranes is essential.

Recent advances in nanotechnology have led to the development of nanoscale selective transducers with excellent sensitivity. Nanosensors developed for plant-related applications include plasmonic nanosensors, Förster resonance energy transfer (FRET)-based nanosensors, carbon-based electrochemical nanosensors, nanowire nanosensors, and antibody nanosensors. These nanoscale sensors allow us to monitor cellular functions, metabolic processes, spatiotemporal changes in plant compounds, and the presence of viral and fungal pathogens (Shaw and Honeychurch, 2022). Additionally, nutrient-coated quantum dots enable the spatiotemporal tracking of organic and inorganic nutrient uptake and movement within plants, even being associated with mycorrhiza (van’t Padje *et al.*, 2021; Raven, 2022).

A further practical output of plant–NM relations may be the integration of nanotechnology and plant biology, leading to the emergence of plant nanobionics, a field which creates plants possessing novel functions such as biochemical sensing or light emission (Lew *et al.*, 2020).

Moreover, seed nano-priming has emerged as a practical, low-cost, and eco-friendly strategy to improve and synchronize germination, promote seedling growth, and induce plant resistance to abiotic and biotic stresses (Pereira *et al.*, 2021; Shelar *et al.*, 2021; Nile *et al.*, 2022). The treatment of seeds with NMs can also be used as a strategy to improve the delivery of nutrients for plant growth and to increment the nutritional quality of food (De La Torre-Roche *et al.*, 2020). Thus, the use of NMs allows improvement in crop production with the application of reduced amounts of pesticides and fertilizers (Pereira *et al.*, 2021).

Different types of NMs can be applied to plants as nano-biostimulants for enhancing photosynthesis, shoot development, and, thus, biomass production (Ranjan *et al.*, 2022), as well as for delaying the post-harvest senescence of ornamental plants and fruits (Naing and Kim, 2020; Seabra *et al.*, 2022). Additionally, NMs have been used in plant *in vitro* cultures as novel elicitors to enhance the synthesis of secondary metabolites, with applications in pharmaceutical, cosmetic, and food industries (Rivero-Montejo *et al.*, 2021). There are other applications of NMs in plant *in vitro* cultures, including the induction of callus production, somatic embryogenesis, organogenesis, biomass accumulation, and flower/fruit production (Khan *et al.*, 2019; Mahajan *et al.*, 2022; Phong *et al.*, 2022).

## Conclusions and knowledge gaps

**Fig. 4.**
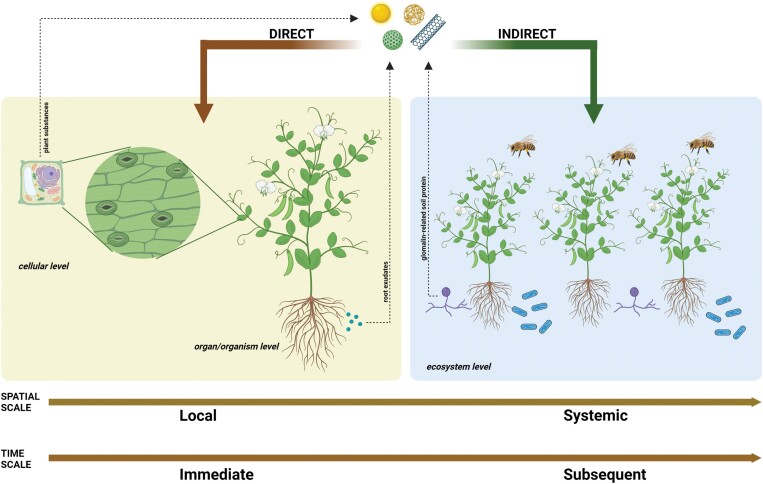
Summary of the multilevel and bidirectional relationship between nanomaterials (NMs) and plants. The NM–plant relationship can be interpreted on spatial and time scales from the immediate, local level (cellular, organ/organism levels) to the subsequent systemic level (ecosystem level). At the cellular, organ, and organism levels, NMs directly affect plants, while at the ecosystem level the effect of NMs is indirect. NMs have an effect on beneficial ecological partners of plant such as plant growth-promoting rhizobacteria, arbuscular mycorrhiza, and pollinators. Substances in plant cells and in root exudates (e.g. amino acids, phenols, and organic acids) influence NM characteristics and availability.

The development of nanotechnology provides an opportunity to use more and more types of NMs in plant cultivation, which may have high relevance from a sustainability point of view. For this we need to have a broad, and at the same time deep, systematic theoretical knowledge of the plant–NM relationship. This review points out that there is a multilevel relationship between plants and NMs which can be interpreted on a spatial scale: from local interactions in cells to systemic effects on whole plants and on ecosystems. The effects of NMs on plants may be immediate or may appear later, meaning that the plant–NM relationships can also be interpreted on a time scale (Fig. 4). The immediate effect is manifested at the cellular level immediately after the NM comes into physical contact with the plant cell; the cell wall- and membrane-modifying impacts of the NMs can be interpreted as immediate effects. Subsequent effects occur after the uptake and translocation of the NM, and these effects affect organs and the whole organism. Furthermore, the influence of NMs on plant ecological partners can be considered as long-term effects. It is also important to understand that, beyond direct effects on plants, NMs also indirectly influence plants as they affect beneficial ecological partners, such as PGPR (including rhizobia), arbuscular mycorrhiza, and pollinator insects. Furthermore, the relationship between NMs and plants is bidirectional, since NMs affect plant development, metabolism, and physiology, and plant substances affect NM availability (root exudates) and characteristics (*in planta* biotransformation, Fig. 4). NMs influence plant physiological processes in every developmental stage during the whole life cycle. In general, the effects of NMs at the organ and organism levels can be considered as hormetic, and the indirect effects of NMs on plant symbionts (especially on PGPR and N fixing) follow the concept of hormesis. This means that hormesis is a general concept when considering NM–plant relationships at different levels.

Despite the fact that the plant–NM interaction is a hot topic in plant biology, the following knowledge gaps were identified.

- Detailed investigation of NM interactions with roots should not focus only on the NM features, but also on the soil constituents and properties.
- In order to gain a more accurate view of the diverse interactions, experimental setups are needed in which the effects of NMs are investigated not only on PGPR in a plant-free system but also in the plant–PGPR system. These studies are important to ensure the safe application of PGPR and NMs as biostimulants in crop production.
- Similarly, experiments should examine plants and their pollinators in a common ecological system, in order to gain valuable information about the consequences of NM toxicity on pollination and plant yield.
- There is a knowledge gap regarding NP accumulation in flowers, nectar, fruits, and seeds, because most of the studies concerned with NP uptake and translocation have been carried out in plants in their vegetative state.
- NM characterization should be performed under realistic conditions (i.e. considering agricultural settings), by evaluating the NM transformations in contact with soil and root exudates.
- Due to the complex composition of root exudates, further studies are required to better understand their impact on NM transformation, uptake, distribution, bioavailability, and fate.
- Additionally, an important goal of future research should be to supplement the current knowledge about the *in planta* biotransformation of various NMs.

The future examination of these partially or barely investigated research areas will allow closer understanding of the complex relationship between NMs and plants.

## Data Availability

No new data were generated in the writing of this review.
